# Use of identification wristbands among patients receiving inpatient treatment in a teaching hospital

**DOI:** 10.1590/0104-1169.0144.2522

**Published:** 2015

**Authors:** Louíse Viecili Hoffmeister, Gisela Maria Schebella Souto de Moura

**Affiliations:** 1 RN; 2 PhD, Adjunct Professor, Escola de Enfermagem, Universidade Federal do Rio Grande do Sul, Porto Alegre, RS, Brazil

**Keywords:** Patient Identification Systems, Patient Safety, Nursing

## Abstract

**OBJECTIVE:**

to evaluate the use of identification wristbands among patients hospitalized in inpatient units.

**METHOD:**

quantitative, descriptive and transversal research, with a sample of 385 patients. Data collection occurred through the observational method through the filling out of a structured questionnaire which aimed to check the presence of the identification wristband and the identifiers used. Descriptive statistics with absolute and relative frequencies was used for analysis.

**RESULTS:**

it was obtained that 83.9% of the patients were found to have the correctly identified wristband, 11.9% had a wristband with errors, and 4.2% of the patients were without a wristband. The main nonconformities found on the identification wristbands were incomplete name, different registration numbers, illegibility of the data and problems with the physical integrity of the wristbands.

**CONCLUSION:**

the study demonstrated the professionals' engagement in the process of patient identification, evidencing a high rate of conformity of the wristbands. Furthermore, it contributed to identify elements in the use of wristbands which may be improved for a safe identification process.

## Introduction

Patient safety has been the focus of discussions over the last decade worldwide. Brazil is part of this mobilization and, since April 2013, has placed emphasis on the actions through the launch of the National Patient Safety Program (PNSP). The program establishes protocols for meeting the international safety guidelines and determines the creation of Patient Safety Nuclei in the health services^(^ 1 ^-^ 2 ^)^.

In order to reduce errors and extend the number of safe practices, health institutions are investing in actions which aim for quality of care and seek to spread a culture of safety for the patients, for the professionals, and for the environment. These changes are focused on the six patient safety goals stipulated by the World Health Organization (WHO), goal number one being the correct identification of the patient^(^ 3 ^)^.

In daily life, it can be observed that the health services adopt different ways of identifying the patients, for example, wristbands, signs on the headboards, stickers on clothes, and identity badges. Since the PNSP was launched, institutions have needed to compatibilize the devices stipulated by the protocol and the patients' wishes. In spite of there existing few studies specifically addressing the issue of patient identification, it is possible to note a worldwide concern in relation to this practice, as it is closely related to any procedures undertaken with the patients, such as the administration of medication, operations, and transfusion of blood or blood products, among others. 

Mistakes in identification can originate back at the moment when the patient is entered in the attendance system. The entering of data with errors, in a computerized record, can compromise the entire care process^(^ 4 ^)^. The process of patient identification, including the checking of data on the wristband against the patient's records and with the information confirmed by him or her, can be seen as an important stage in the interaction between the patient and the health team. Errors caused by carelessness can continue to occur if the patients do not have wristbands, or if the wristband does not contain accurate information for identification^(^ 5 ^)^.

In 2007, the National Patient Safety Agency (NPSA) of England and Wales revealed in a publication that more than one in 10 cases of incompatible care measures notified were related to wristbands. The correct use of wristbands and the definition of standards regarding color, material and identifiers promote safe practice regarding patient identification, which means an additional resource in the combating of the devastating errors which incorrect identification can bring^(^ 6 ^)^. Following the provision, by the NPSA, of guidance for safe practice related to identification, it was observed that 98% of the hospitals had developed policies which were consistent with these directives, although 23% reported difficulties in implantation and in adherence by patients and teams^(^ 7 ^)^.

The identification of the patient has two purposes: firstly, to safely determine the individual as being the legitimate receiver of the treatment or procedure; secondly, to ensure that the procedure to be undertaken is effectively that which the patient needs^(^ 8 ^)^. In day-to-day practice, one can perceive that patient identification is a stage of the nursing care which does not receive the appropriate attention, although it can influence the other stages and is essential in order to ensure the quality and safety of the service provided. 

Considering the aspects raised regarding the importance of the correct identification of the patient and its relationship with the occurrence of errors in healthcare, and, on the other hand, the extent to which this is little valued in the practice environments, the following research question arose: are the patients using wristbands which ensure their correct identification? As a result, the present study was undertaken with the aim of assessing the use of the wristband among patients hospitalized in inpatient units in a teaching hospital.

## Method

This is a study with a quantitative, descriptive and transversal approach, undertaken in a teaching hospital with 841 beds, in Porto Alegre in the state of Río Grande do Sul, Brazil^(^ 9 ^)^. The data were collected in the clinical, surgical, mother and child and pediatric inpatient units, totaling 19 departments.

The study population was the patients hospitalized in the above-mentioned units. The sample was defined non-probabilistically, with the patients being selected by convenience. In 2011, the hospital obtained a total of 22,112 episodes of inpatient treatment in these units^(^ 10 ^)^. In order to calculate the proportion of correct use of the identification wristband in such a way as to maximize variance (estimating that 50% would be using the wristband correctly), considering a margin of error of five percentage points and the level of confidence of 95%, it was necessary to have 385 cases. The 385 cases were selected respecting the proportionality of the bed spaces of the above-mentioned units in the total of the bed spaces in the hospital. 

As an inclusion criteria, the patient's inpatient treatment in the above-mentioned hospital departments was adopted. The following were excluded: patients in outpatient consultations, patients of areas providing diagnosis and therapeutic sessions, outpatient surgical patients, emergency patients, patients from the surgical center, patients from the intensive care center, and patients from the obstetric center, as well as those patients who for whatever reason could not sign the terms of consent. 

Data collection took place during five consecutive days in November 2012 through the observational method, through the filling out of a structured questionnaire which covered data referent to the use of, and conditions of, the wristband, the identification elements used in the wristband, and the identification data found in the patient's hospital records. The data from the wristband were noted in the research instrument for later checking against the data found in the patient's hospital records. The nurses responsible for the units were advised when it was observed, after data collection, that the patients were without wristbands or when there was some irregularity in the data or in the wristband's conditions. Each inpatient department was evaluated only on one occasion, and without prior arrangement. 

As it involved categorical variables, for analysis of the data, the researchers used descriptive statistics with absolute frequencies (n) and relative frequencies (%). The data were stored and analyzed using the SPSS software, version 16.0. 

The study was undertaken following the approval of the Ethics Committee of the Research and Postgraduate Group of the above-mentioned hospital, under number 1203-84. The obtaining of the data from the hospital records occurred through the signing of the terms of consent for data use, which was standardized in the hospital, by the researcher responsible for the study. For the collection of the data, the participants - aged over 18 years old - were requested to read and sign the terms of consent. Specific terms of consent were created for patients who were minors, to be signed by their parents or guardians. 

## Results

The present study ascertained that of the 385 patients observed, 369 (95.8%) had the wristband, and 16 (4.2%) patients did not have a wristband. Of the patients with the wristband, it is observed that 83.9% (323) were identified in accordance with what is stipulated by the institution's Standard Operating Procedure (SOP) for patient identification, while 11.9% were not in accordance. The conformities were related to the presence and physical integrity of the wristband, the legibility of the data, the presence of two identifiers - these being the complete name and the number of the hospital records - and to the data found on the wristband being in accordance with the data found in the patient's online hospital records.

Among the cases of patients who had the wristband but who were not in conformity with the institution's nursing SOP, one can highlight the errors related to the non-integrity of the wristband, illegibility, and inconsistencies between the name written on the wristband and the name found in the online hospital records, and the patient's registration number found on the wristband compared to the registration number of the online hospital records. 

In relation to the wristband's physical integrity, only 3 (0.81%) wristbands of the 369 were found to have problems in relation to physical integrity, due to having rips (two wristbands) and folds (one wristband). 

In relation to the legibility of the data on the wristband, it was found that one wristband had the name and hospital number unclearly printed on the sticky label glued on the wristband, one wristband had just the registration number unclearly printed on the sticky label, and in the case of two wristbands which had the name unclearly printed, it had been written directly onto the wristband (without the use of a sticky label as in the other cases).

In relation to the fact of the name written on the wristband being in accordance with the name found in the electronic hospital records, irregularities were found in 32 (8.67%) wristbands, with 25 of these having the incomplete name of the patient, 2 having the wrong surname, 2 having misspellings of the patient's first name and/or surname, and 3 with the first name incomplete and the wrong surname. 

In relation to the fact of the registration number written on the wristband being in accordance with the number found in the electronic hospital record, it was ascertained that 16 (4.33%) wristbands had errors. Among these, 3 wristbands had one digit wrong; and 9 wristbands had an entirely different number, that is, there was no agreement between the registration number in the records and that on the wristband. It was also seen that in 3 wristbands it was not possible to read some numbers, and that in one wristband there was no registration number. 

The color of the wristband was also a variable investigated, the use of 278 (75.3%) white wristbands and 91 (24.7%) orange wristbands, which indicated the presence of some form of allergy, being observed. In some patients, it was observed that their wristbands were covered by anti-allergenic micropore, as they had developed allergies to the material of the wristband. The alternative, to cover the wristband, was found by the nursing team so that the patient would not have to forgo the use of the wristband. 

The institution's SOP for patient identification establishes that the wristband must contain, at the minimum, two identifiers - the complete name of the patient and the registration number. The bed number, in this hospital, is not considered a reliable identifier. Of the 369 patients who have the wristband, 364 (98.64%) wristbands were identified with two identifiers and 5 (1.36%) wristbands had three identifiers. In relation to the type of identifiers, it is possible to identify that 363 (98.38%) had, as the identifiers, the name and registration number, one (0.27%) had the name and the bed number, 4 (1.08%) wristbands had to the name, registration number, and bed number and - furthermore - one (0.27%) had the name, registration number, and medical team. 

In relation to the 16 patients who were without a wristband, 6 (37.5%) cases occurred in the pediatric inpatient unit. The main reason described by the children's parents or guardians was that, when they attached the wristband, the nursing professionals left it too large for the size of the children's forearms, leading to the loss of the wristbands. A further 4 (25%) patients without wristbands were receiving inpatient treatment in the psychiatric unit, it being the case that the patients themselves reported that they had no wristband because they did not want to use them. The 6 (37.5%) remaining patients were receiving inpatient treatment in the adult clinical and surgical inpatient units. On being questioned as to why they had no wristbands, 2 of them responded that they did not want to use them and that they did not even believe that this practice was important, while the other 4 stated that the wristband had been removed by the nursing team in order to place a venous access point, and that the team had forgotten to replace it on the other arm. 

## Discussion

According to the results presented, 83.9% (323) of the patients were identified in accordance with the requirements described in the institution's nursing SOP. To monitor the proportion of patients using the standardized wristband is one of the practices recommended in the patient identification protocol^(^ 2 ^)^.

Although the percentage evidenced seems to be an excellent result, the number of individuals identified correctly should be close to 100%, mainly because the identification of the patients is an important stage which precedes the majority of care measures. In another study, the authors recommend that the error rate, relative to wristbands, should be kept at between 0.2% and 0.3%^(^ 11 ^)^.

Even though it did not achieve the ideal values in the correct implementation of the wristbands, the institution in which the data were collected presents significant results when compared to data from other institutions. In one study undertaken in a hospital in São Paulo, Brazil, 540 observations were undertaken in relation to the wristbands on neonates receiving inpatient care and a total of 82.2% of conformity was obtained in the wristbands, in accordance with the institution's protocol. In relation to the nonconformities, the most frequent were the presence of the incomplete name of the mother of the newborn (6.7%), and the illegibility of the data on the wristband (6.9%)^(^ 12 ^)^.

One American study, which brought together data from 217 health institutions participating in research in the years 1999 and 2000, obtained a total of 1,757,730 wristbands evaluated. Of this total, 45,197 (2.57%) of the observations contained errors,: 71.6% were attributed to absent wristbands, 7.7% to illegible wristbands, 6.8% to wristbands with incorrect information, 9.1% to wristbands with unclearly printed information, 3.7% to wristbands which had conflicting data, and 1.1% in which the wristband was wrong^(^ 11 ^)^, demonstrating that the problem of the absence and of the illegibility of the wristbands was the most frequent issue. The authors also mentioned that wrong wristbands are easier to correct than absent wristbands, which supports the results of the present study, in which the rate of individuals without a wristband is lower than the rate of individuals with wrong wristbands.

In the same study, published by the College of American Pathologists, the authors state that requiring all patients always to use a correct wristband does not require a broad effort with expensive equipment and elaborate systems, but, rather, requires the strengthening of the simple system already in place^(^ 11 ^)^. In consonance with this, in one Brazilian study, the authors confirmed that "while the equipment for the use of barcodes can have a high cost, the use of wristbands and the appropriate identification on the bed generate lower costs and, if used effectively, contribute to minimizing the occurrence of the administration of medications to the wrong patients"^(^ 13 ^)^.

The absence of the wristband and errors in the patient's name and identification number are recognized as the most frequent types of error when one is discussing the checking of patients' wristbands. One study conducted over 45 months indicated that the absence of wristbands is responsible for 50.3% to 100% of the problems found, the name for 0% to 24.6%, and the number for 0% to 25.3% of the errors^(^ 14 ^)^.

The strategy of implanting wristbands as one of the tools for promoting the care which strives for the patients' safety is configured as a low-cost practice for the institutions, which is easy to install in the health professionals' routine of care.

The fact that the nursing professionals have to write the patients' data on the wristbands is configured as a point at which failures can occur, when one takes into consideration these professionals' high workload in various institutions. The implantation of new technologies is described in the literature as an alternative for the safer identification of the patient. Researchers state that the use of barcodes on the wristbands and of a scanner (for the reading of the same), prior to the administration of medications, constitutes a means of ensuring that the medication prescribed is being administered to the correct patient, estimating a reduction of approximately 70% in the error rates of institutions which use this system^(^ 13 ^)^. One recent study ratifies the wide use of wristbands with barcodes and the reduction of the number of errors related to this stage of the care; however, it also considers that the disadvantage of this technology is the cost of its implantation^(^ 15 ^)^.

It is calculated that the costs of implanting barcode technology in wristbands is between US$200,000 and US$1 million, depending on the size of the hospital^(^ 15 ^)^. In comparison, a study undertaken in the United States indicates that 1% to 2% of patients receiving inpatient treatment suffer harm resulting from medication errors, and describe that each error results in an additional cost of US$4,700 to US$5000, without taking into account the legal costs^(^ 16 ^)^. In analyzing these data, and those from another study, which described that of 24,382 errors recorded, 2900 were related to patient identification, one can calculate a cost of over US$ 13 million in order to fix such errors^(^ 6 ^)^. In this perspective of analysis, this technology's implantation would represent a lower cost for the institutions, and safer care. 

It is appropriate to warn that, even with the incorporation of the technology, it is necessary for the professionals to rigorously follow the recommendations to scan the barcodes on the patient's wristband and on the medication to be administered, in order to confer safety on the process. A study undertaken in hospitals reveals that, sometimes, these care steps are not followed due to the wristbands being damaged (wet from body fluids, crumpled or torn) and to the data being illegible, or even inaccessible due to the patient being asleep^(^ 17 ^)^.

The results evidence that the largest number of patients without wristbands was found in the pediatric units and in the psychiatric unit. In the light of this, one should emphasize the importance of giving greater attention to the identification of patients receiving inpatient treatment in these units. In one Brazilian study published in 2011, the researchers undertook observations of the process of administering medications and fluids, it being the case that in 36.32% of these, the pediatric patients were not identified in any way, and that in 63.67% of cases, these patients were identified in some way^(^ 18 ^)^. Another study reinforces the need for greater attention regarding this group due to the fact that pediatric patients have barriers to verbal communication, requiring the active participation of family members in confirming identification^(^ 19 ^)^. The wristband, for pediatric use, must have a minimum size so as to ensure comfort and safety for this special group of patients^(^ 2 ^)^.

In relation to the psychiatric inpatient unit, the single reason provided by the patients themselves referent to the absence of the wristband was that they had no interest in using the wristbands stipulated by the hospital. In the search for references which might help in understanding this result, a single study was found which raised data on psychiatric units. In it, the authors present that of 14 patients observed, 14 did not have the wristbands - that is, 100% of the patients - and that, when the nurse responsible for the unit was questioned, she stated that the reason for this result was the small number of beds, which led the team to know all the patients receiving inpatient treatment^(^ 20 ^)^.

Attention is drawn to the fact that even in units with few beds, one must take into consideration that the patients receiving inpatient treatment are attended by various teams made up of different professionals, who on most occasions are providing care to these patients during only one part of the day. This situation also occurs with staff from other departments, not only at the time in which one professional appears in the inpatient unit in order to collect blood for tests, but also when one professional sees and attends a patient in an area outside the unit. The use of the identification of the patient becomes necessary in the provision of care by the team which has the greatest contact with the patient and by the other staff and students, as well as its importance being fundamental when the patient is transferred by the hospital. As a result, the dissemination of awareness of the relevance of the use of wristbands by both the care teams and the patients themselves is essential, such that these may become co-responsible for this practice. 

The color used on the wristbands was also one result found. The majority of the patients made use of white wristbands, although the percentage (24.7%) of patients with orange wristbands as an indication of an allergy was relevant. The use of colors in wristbands placed on the patients' forearms has been a strategy adopted by the institutions in order to indicate some specific characteristic in the health situation of patients receiving inpatient treatment, which require greater attention on the part of the teams which attend them. The institutions undertake this practice without appropriate standardization between them, it being noted that in some hospitals the orange wristbands indicate allergy, while in others, they indicate risk of falls. In one initiative of the "Colorado Foundation for Medical Care", the managers of the hospitals of this region of the United States met in order to discuss and define a standardization for the colors of the wristbands, following a case of a nurse classifying a patient inappropriately, in which the color defined for allergy was red^(^ 21 ^)^.

A recent publication emphasizes that the absence of standardization can confuse those professionals who work in more than one institution, in relation to the color of the wristband^[1]^. Thus, in Pennsylvania (USA), the standardized use of colors for indicating the risk of the occurrence of safety events was proposed^(^ 22 ^)^.

In relation to this issue, the Brazilian Network for Nursing and Patient Safety - Rio Grande do Sul Branch (REBRAENSP - RS) held discussions on the standardization of wristband colors, with the aim of defining the most appropriate color for indicating allergy. After the reports from nurses from different institutions, it was ascertained that the colors used most were orange and red. In a recent publication, the Network chose to suggest only the use of the color red, in order to reduce still further the scope for possible mistakes^(^ 23 ^)^.

Adopting improved practices and new working routines in the health institutions is a complex process, due to the time the teams take to adapt to new policies. The already-established routines ensure a comfort zone for the professionals, while the proposal to reconfigure the actions can trigger feelings of insecurity, rejection and fear. The difficult task of implanting new routines has already been described in a previous study, it being recognized that policies which aim to change the behavior of the practitioners in order to improve safety are less likely to be successful if they do not take the pre-existing practices into account^(^ 5 ^)^.

The identification of the patients, and the application of the wristband, must occur in a systematized process, this action being included as one of the measures of care provided to the patients. This fact can be observed in analyzing recent data referent to the neonatology inpatient treatment unit, where 100% of the newborns have at least one wristband, and 100% of the wristbands are in accordance with the SOP. The practice of placing wristbands on the newborns, and confirmation of the data on the wristbands with those responsible for the child has been absorbed by this unit's professionals, as is the case with the other care measures undertaken in admitting newborns. 

The process of implanting new practices must take place collaboratively and constructively, allying the institutional objective of consolidating care with quality and safety with the interests and needs of the teams who work in the frontline of the care. Continuing education, updating, improvement and refresher courses with the aim of adding to the knowledge acquired in the basic curricular training also help in reducing failures^(^ 24 ^)^. Another study notes that the team must be involved for there to be understanding, valuing and awareness of the relevance of patient identification, and that the participation of the nursing staff is of fundamental importance in defining the strategies which will be effective in implanting and improving the practices. In order to guarantee patient safety, it is necessary to raise the awareness of all the professionals that the provision of appropriate care, causing no harm, is a responsibility not only of nursing, but of all the teams which interact with the patient^(^ 25 ^)^.

It is considered that one limitation of this study is the fact that it was undertaken at that particular time at the institution, characterized by the implementation of a high number of new routines, and by the turmoil of the process of Hospital Accreditation, which may suggest an increase in the rate of patients identified correctly. 

Another consideration to be made is related to this study's objectives, as it proposed to analyze one of the aspects of identifying the patients: the presence of the wristband on the patients' forearms, and the accuracy of the information inserted in these wristbands. The undertaking of studies which seek to investigate the use of the wristband by the professionals of the institution prior to the provision of care is also necessary in order to assess the process of identification as a whole.

## Acknowledgments

To Hospital de Clínicas de Porto Alegre (HCPA) by agreement in conducting the research and statistical support of the Grupo de Pesquisa e Pós-Graduação (GPPG / HCPA).

## Conclusion

Through this study, it was possible to investigate the prevalence of the use of wristbands in patients receiving inpatient treatment in a teaching hospital in the municipality of Porto Alegre, Río Grande do Sul, Brazil. This practice's implementation was verified by the rates of patients with the wristband and by the rates of correct wristbands in accordance with the institution's SOP. It was ascertained that the use of the two identifiers, the complete name and registration number, is widely spread among the professionals who insert these data in the wristbands. Data were also found which brought results referent to the visual aspects of the wristband, such as its physical integrity, its legibility, and its color. Furthermore, the inpatient units which require greater attention, due to presenting results below those found in the majority of areas investigated, were identified. 

The results obtained in the units of the pediatric and psychiatric services are highly similar to the references available in the literature, which reinforces the need for a review of the stages of identification with the health team, and a raising of the patients' and their family members' awareness regarding the importance of these being alert to this practice in participating in the construction of the culture of safety in the institution. 

The nonexistence of similar, pre-existing research makes it impossible to evaluate the influence of the current situation of accreditation in comparison with the period prior to the spreading of ideas of safety and quality. The present study demonstrated the professionals' engagement in the process of identifying patients, as it verified a high rate of conformity in the wristbands observed with the institution's SOP, even though this had been created less than one year previously, intending to encompass the international safety recommendations. 

This study's undertaking contributed through providing evidence of the use of wristbands in patients receiving inpatient treatment in the institution, and guiding the improvement of the practices referent to this issue. It is suggested that monitoring should be undertaken of this process some months after the evaluation of the Joint Commission International, so that the progress of the actions may be visualized, and that comparisons may be made between the different time periods experienced by the hospital, making it possible to assess the consolidation of a culture of safety in the institution. 
